# TACI Contributes to *Plasmodium yoelii* Host Resistance by Controlling T Follicular Helper Cell Response and Germinal Center Formation

**DOI:** 10.3389/fimmu.2018.02612

**Published:** 2018-11-09

**Authors:** Marcela Parra, Jiyeon Yang, Megan Weitner, Steven Derrick, Amy Yang, Thomas Schmidt, Balwan Singh, Alberto Moreno, Mustafa Akkoyunlu

**Affiliations:** ^1^US Food and Drug Administration, Division of Bacterial Allergenic and Parasitic Diseases, Center for Biologics Evaluation and Research, Silver Spring, MD, United States; ^2^Emory Vaccine Center, Yerkes National Primate Research Center, Emory University, Atlanta, GA, United States; ^3^Division of Infectious Diseases, Department of Medicine, Emory University School of Medicine, Atlanta, GA, United States

**Keywords:** *Plasmodium yoelii*, TACI, T follicular helper cell, Germinal center, B cell, antibody

## Abstract

The delay in parasite-specific B cell development leaves people in malaria endemic areas vulnerable to repeated *Plasmodium* infections. Here, we investigated the role of transmembrane activator and calcium-modulator and cyclophilin ligand interactor (TACI), a molecule involved in the generation of antigen-specific antibody secreting cells, in host response to non-lethal *Plasmodium yoelii* infection. We found that TACI deficiency not only resulted in higher peak parasitemia levels in *P. yoelii* challenged mice, but also led to a delay in parasite clearance and anti-*P. yoelii* Merozoite Surface Protein 1(C-terminal 19-kDa fragment [rMSP-1_19_]) protein and anti-rMSP-1_19_ and anti-*P. yoelii* IgG antibody development. There was also a delay in the generation of splenic high affinity antibody secreting cells that recognize rMSP-1_19_ protein as compared to wild-type mice. Interestingly, coinciding with the delay in parasite clearance there was a delay in the resolution of T follicular helper (T_FH_) cell and germinal center (GC) B cell responses in TACI -/- mice. The persistence of T_FH_ and GC B cells is likely a result of enhanced interaction between T_FH_ and GC B cells because inducible costimulator ligand (ICOSL) expression was significantly higher on TACI -/- GC B cells than wild-type cells. The difference in the kinetics of GC reaction appeared to also impact the emergence of plasma cells (PC) because there was a delay in the generation of TACI -/- mice PC. Nevertheless, following the recovery from *P. yoelii* infection, TACI -/- and wild-type mice were both protected from a rechallenge infection. Establishment of protective B cell response was responsible for the resolution of parasitemia because B cells purified from recovered TACI -/- or wild-type mice were equally protective when introduced to naïve wild-type mice prior to *P. yoelii* challenge. Thus, despite the increased susceptibility of TACI -/- mice to *P. yoelii* infection and a delay in the development of protective antibody levels, TACI -/- mice are able to clear the infection and resist rechallenge infection.

## Introduction

Children under the age of 5 years (1), especially those who are less than 1 year of age, are highly vulnerable to *Plasmodium* infections (2). While antibodies play a critical role in controlling parasitemia burden and illness (3), protective humoral immunity to malaria occurs only after repeated exposure to parasites (4). Shortcomings of immunological response that can control *Plasmodium* parasites have been attributed to the diversity of the malarial antigens, the rapid disappearance of anti-malarial antibodies and an insufficient long-lived plasma cell (PC) pool (4). Despite the recognition of these B cell insufficiencies, molecular and cellular events that prevent the host's ability to mount optimal B cell responses are poorly understood.

In this study, we examined the role of transmembrane activator and calcium modulator and cyclophilin ligand interactor (TACI) in host resistance to malaria infection. TACI is a receptor for B cell activating factor belonging to TNF family (BAFF) and a proliferation-inducing ligand (APRIL) (5). Together with two other receptors, BAFF receptor (BAFF-R) and B cell maturation antigen (BCMA), these molecules are crucial in maintaining B cell homeostasis, and TACI is involved in immunoglobulin isotype switching and antibody secretion, PC maintenance and macrophage polarization (6–10). TACI is also important in controlling T follicular helper (T_FH_) cell responses as immunization or infection of TACI deficient mouse results with augmented T_FH_ development (11, 12). However, while immunization of TACI -/- mice with a T cell dependent antigen elicited reduced antibody responses and short lived PC as compared to wild-type mice (11), TACI -/- mice controlled *Citrobacter rodentium* infection better than the wild-type mice most likely because of an increase in antibody secreting cells and development of high affinity antibodies directed against *C. rodentium* (12). Measurement of elevated circulating BAFF and increased BAFF-R on B cells in humans experimentally challenged with *Plasmodium falciparum* suggest an involvement of these molecules in host response to malaria (13, 14). Whether TACI participates in BAFF-induced host responses during malaria infection has not been explored.

We found that *Plasmodium yoelii* challenged TACI -/- mice manifested significantly higher levels of parasitemia than wild-type mice, which persisted longer. The increased susceptibility of TACI -/- mice appeared to be the result of a delay in anti-parasite antibody development. Analysis of T_FH_ cell development and germinal center (GC) formation suggested that altered kinetics of GC reaction may be responsible for the delay in the PC development and antibody production in infected TACI -/- mice. Nevertheless, despite late parasite clearance, not only were the TACI -/- mice protected from a second *P. yoelii* challenge, but also, B cells from TACI -/- mice were sufficient to prevent *P. yoelii* infection when transferred to naïve wild-type mice. In the absence of TACI, host control of parasitemia is delayed compared to wild-type mice. However, once the parasitemia is cleared, B cell mediated immunity renders TACI -/- mice resistant to a second infection.

## Materials and methods

### Mice

C57BL/6 mice (6–8 weeks old) were purchased from the Jackson Laboratories (Bar Harbor, ME). TACI -/- mice on a C57BL/6 background were described previously (15, 16). The experimental procedures were approved by the Institutional Animal Care and Use Committee of the Center for Biologics Evaluation and Research (Protocol-2008-08).

### Parasites

Nonlethal *P. yoelii* strain 17XNL was used in mouse challenge experiments (17). Frozen stocks of *P. yoelii* 17XNL-infected erythrocytes were intraperitoneally (i.p.) injected to C57BL/6 mice to generate donor mice. When 8 to 10% parasitemia was detected, blood was collected by cardiac puncture, diluted in PBS and used to i.p. infect experimental animals with 1 × 10^6^ *P. yoelii* parasites in 200 μl of PBS. The percent parasitemia [parasitized red blood cells (RBCs)/total RBCsx100] after infection was determined by Giemsa-stained thin blood smears.

### Anti-plasmodium antibody detection

Serum samples were pooled or not from 3 C57BL/6 and 3 TACI -/- mice per time point at 8, 16, 22, 28, and 71 days post *P. yoelii* infection. Serum antibody levels against an extended version of the *P. yoelii* Merozoite Surface Protein 1(C-terminal 19-kDa fragment [rMSP-1_19_]) (18) and whole *P. yoelii* 17XNL extract were measured by ELISA. ELISA plates were coated with 70 ng/well of rMSP-1_19_ or sonicated *P. yoelii* infected RBCs in coating buffer (15 mM Na_2_CO_3_ and 35 mM NaHCO_3_). After washing with PBS/0.05%Tween-20, plates were blocked with 5% milk/PBS. Next, 100 μl of 1:50 to 1:51200 titrated sera were added. After 2 h, plates were incubated with 1:3500 diluted goat anti-mouse IgG-HRP antibody (Southern Biotechnology Associates, Birmingham, AL). Plates were read on a VERSA max microplate reader after adding ABTS substrate (Molecular Devices, Sunnydale, CA).

### Adoptive transfer of B cells

Splenic B cells of mice that had cleared *P. yoelii* infection 4 months earlier were isolated by using B220 magnetic beads (Miltenyi Biotec, San Diego, CA). The purity of B220^+^CD19^+^ B cells was > 95%. *Plasmodium yoelii* immune B-cells were then adoptively transferred by intravenous (i.v.) injection of 3 × 10^7^ immune cells per mouse. Two hours after the B cell transfer, mice were injected with *P. yoelii* infected RBCs. The percent parasitemia (parasitized RBCs/total RBCs × 100) after infection was determined by examining Giemsa-stained thin blood smears.

### Flow cytometry

Single cell suspensions prepared from spleen and dead cells were excluded after staining with fixable efluor 780 (Affymatrix, Santa Clare, CA). For T_FH_ analysis, splenocytes were stained in 2%FBS/0.5 EDTA/PBS buffer with anti-CD4-PercpCy5.5 (clone GK1.55, Affymatrix), PD-1-PE (clone 29F.1A12, BioLegend, San Diego, CA), CD44-Alexa Fluor 700 (clone IM7, BD Biosciences, San Jose, CA), CXCR5-biotin (clone 2G8, BD Biosciences), and ICOSL-biotin (clone HK5.3, Biolegend) antibodies. Biotin was detected with streptavidin-BV421 (BioLegend). For GC B cell and PC analysis, B220-BV605 (clone RA3-6B2, BioLegend), FAS-APC (clone J02, BioLegend), T/B cell activating antigen-FITC (clone GL-7, BioLegend), B220-APC (clone RA3-6B2, BioLegend), CD138-PE (clone 281-2 from BioLegend), and IgD-BV605 (clone 11-26c.2a, BD Biosciences) antibodies were used. For intracellular staining, samples were fixed with the Foxp3 Fix/Perm buffer set, following the manufacturer's instructions (eBioscience). Samples were then intracellularly stained with α-Foxp3 (BioLegend, 150D, 1:100) antibody. 'Fluorescence minus one' (FMO) controls were used to gate the cells for each antibody. Data were acquired on LSRII flow cytometer (BD Biosciences) and analyzed using FlowJo software v10 (FlowJo, Ashland, OR).

### Immunofluorescence microscopy

OCT-embedded spleens were snap-frozen by floating on liquid nitrogen-cooled isopentane. Frozen tissues were cut into 10 μm sections using a cryostat and mounted on poly-lysine-coated slides. Sections were allowed to air dry at room temperature for 10 min and fixed with pre-cooled Acetone and Methanol (1:1 vol) for 10 min, followed by washing three times with PBS containing 0.5% Tween-20. Sections were first blocked with 5% goat serum for 1 h, and then stained overnight at 4°C with anti-B220 (Rat IgG, 1:50, eBioscience), anti-GL-7 (Rat IgM, 1:50, BioLegend) antibodies. Following three washing steps with PBS containing 0.5% Tween-20, sections were stained for 1 h at room temperature with the following secondary antibodies: goat anti-rat IgG-AF488, goat anti-rat IgM-AF647 (1:200, Invitrogen, Carlsbad, CA). Samples were imaged on Leica DMI6000 Inverted Light Microscope. The tile scan function was used to stitch individual 10x images together.

### Elispot assay

rMSP-1_19_-specific antibody secreting cells (ASC) were quantified by direct *ex vivo* ELISPOT assay. Ninety-six-well nitrocellulose plates (Multiscreen-HA; Millipore, Bedford, MA) were coated overnight at 4°C with rMSP-1_19_ at 10 μg/ml concentration and incubated in 15 μM 2-mercaptoethanol/10%FBS/RPMI for 1 h. Splenocytes or bone marrow cells (10^6^ cells/well/100 μl) were incubated at 37°C, 5% CO_2_ for 5 h in RPMI medium. Plates were washed, and bound rMSP-1_19_-specific IgG-producing cells were stained with goat anti-mouse IgG-HRP (Bethyl Laboratories, Montgomery, TX) antibody. HRP was developed with 3-aminoethyl carbazole, AEC peroxidase substrate kit (Vector Laboratories, Burlingame, CA). Spots were counted using an AID ELISPOT analyzer (Autoimmun Diagnostika, Germany).

### Antibody avidity measurement

Serum samples collected from 5 C57BL/6 mice and 5 TACI -/- mice 71 days after *P. yoelii* infection were pooled. The avidity of the antibodies was evaluated using guanidine hydrochloride (GuHCl) as a dissociative agent (19). The ELISA plates were coated, blocked and serum titrations were prepared as described in ELISA method. Triplicate serum samples were added to each well. After 2 h of incubation and washing steps, “avidity samples” were incubated with 100 μl of 0.1 M GuHCl (Sigma, Darmstadt, Germany) while triplicate “control samples” were incubated in washing buffer. After incubation for 10 min and 5 washes, wells were exposed to goat anti-mouse IgG-HRP antibody (Southern Biotechnology Associates) for 1 h. Following washing steps, ABTS substrate was added and the plates were read on a VERSA max microplate reader (Molecular Devices). Antibody avidity was calculated using the method described by Perciani et al. (20). Optical densities from each titration were graphed using GraphPad Prism software (La Jolla, CA) and the area under the curve (AUC) was measured for both the GuHCl and control-treated samples for each serum pool. The formula (AUC of guanidine treated samples)/(AUC of control-treated samples) was used to calculate the avidity index ratio.

### Statistical analyses

The parasitemia data were evaluated using unpaired Student's *t*-test. Unpaired Student's *t*-test was also used for the comparison of cell numbers measured in flow cytometry and ELISPOT analysis. Values of *p* < 0.05 were considered statistically significant. The avidity ELISA data was evaluated using AUC with a baseline value of 0, and the means compared using a Mann-Whitney non-parametric test.

## Results

### Elevated parasitemia and delayed clearance of *p. yoelii* in TACI -/- mice

**Figure 1:**
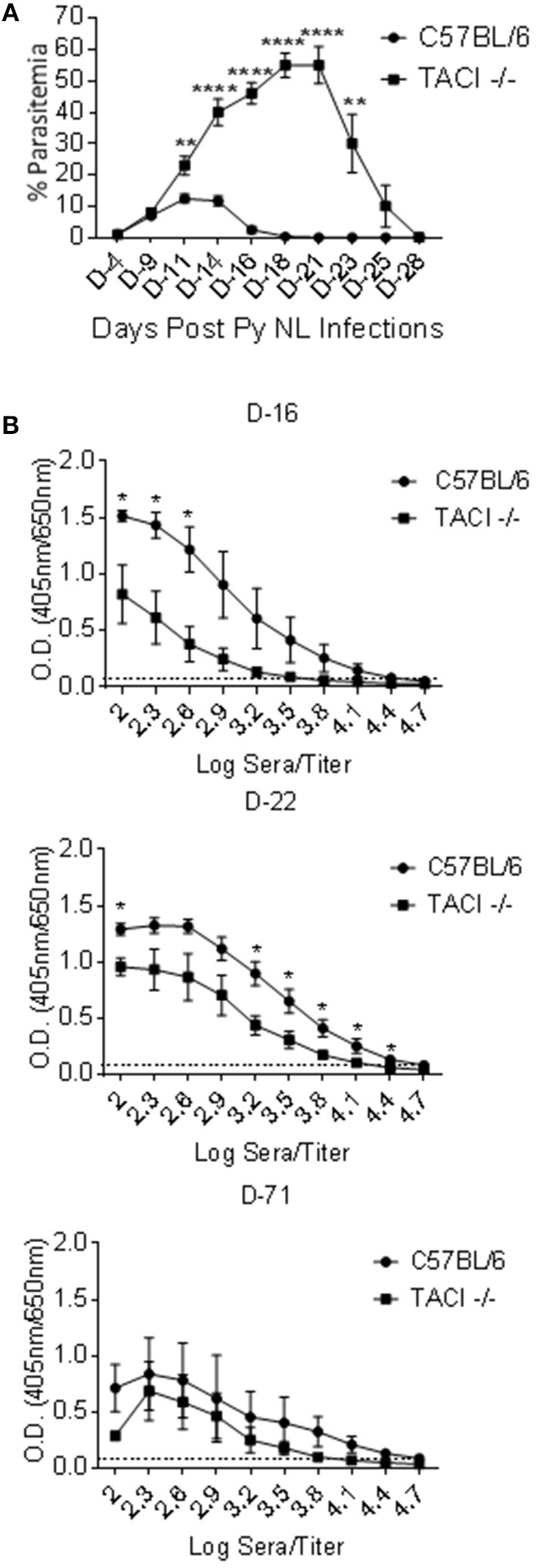
*P. yoelii* infection clearance and antibody development are delayed in TACI -/- mice. **(A)** TACI -/- and C57BL/6 mice were infected (i.p.) with 1 × 10^6^ *P. yoelii* (Py 17XNL) parasites. Parasitemia levels were evaluated by blood smears at indicated days post challenge until parasitemia clearance. Results are expressed as mean % parasitemia ± SEM for 10 mice per group. One representative experiment out of three with similar infection outcome is shown. **(B)** TACI -/- and C57BL/6 mice (9 per group) were infected (i.p.) with 1 × 10^6^ *P. yoelii* parasites. Individual serum was collected from three mice of each group (per time point) on days 16, 22, and 71 post parasite challenge and were tested in ELISA for anti-rMSP-1_19_ IgG antibody measurement (Please see Supplementary Figure 1A for days 8 and 28 ELISA results). Average OD values ± SEM from three samples on each time point were plotted. Dotted line indicates ELISA negative control. Unpaired student's *t*-test was used for statistical evaluation. *indicates *p* < 0.05, ** < 0.01; ****p < 0.0001. Experiment was performed once.

To assess whether TACI participates in resistance to *Plasmodium* infection, we challenged C57BL/6 and TACI -/- mice with *P. yoelii*. Parasitemia levels were monitored by counting the percentage of *P. yoelii* infected RBCs until the resolution of parasitemia. The wild-type mice developed a typical self-limiting course of parasitemias that characterize infections with the non-lethal *P. yoelii* strain 17XNL (21), with peak parasitemia on day 11 post-infection, and parasitemia resolution by day 21 post-infection (Figure 1A). In contrast, parasitemia levels were significantly higher in TACI -/- mice starting at day 11 post-*P. yoelii* infection when compared to the wild-type mice. Parasitemias continued to increase in TACI -/- mice after day 11 post-infection, until reaching a peak at day 21 post-infection. In contrast, C57BL/6 mice had 0% parasitemia at day 21 post-infection. Thus, in the absence of TACI, malaria parasite load was increased, and parasitemia resolution was markedly delayed.

### Anti-*plasmodium* antibody responses are delayed in TACI -/- mice

Antibodies to *Plasmodium* are involved in reducing parasite load and clearance in both mice and humans (3, 22). To assess whether antibody development was altered in parallel to the delay of parasitemia resolution in TACI -/- mice, serum antibodies against rMSP-1_19_, a surface exposed fraction of *P. yoelii* MSP-1 protein that is a target of protective antibodies (18), were measured over the course of the infection. Both strains had low levels of anti-rMSP-1_19_ IgG antibodies on day 8 post-infection (Supplementary Figure 1A). Although anti-rMSP-1_19_ IgG levels increased thereafter in both strains, wild-type mice levels were markedly higher on days 16 and 22 as compared to those of TACI -/- mice (Figure 1B). TACI -/- mice antibody levels reached comparable levels to those elicited in the wild-type mice only on day 28 (Supplementary Figure 1A) and remained at levels comparable to those of wild-type mice at 71 days post infection (Figure 1B). Similar trends in antibody responses were observed when sera were tested for IgG antibodies against *P yoelii* parasites (Supplementary Figure 1B). Since the peak anti-*P. yoelii* antibody development coincided with the parasitemia resolution time-point in TACI -/- mice, this delayed antibody response is likely responsible for the magnitude and the delay in the resolution of parasitemia.

### TACI deficiency extends formation and resolution kinetics of the GC response

**Figure 2:**
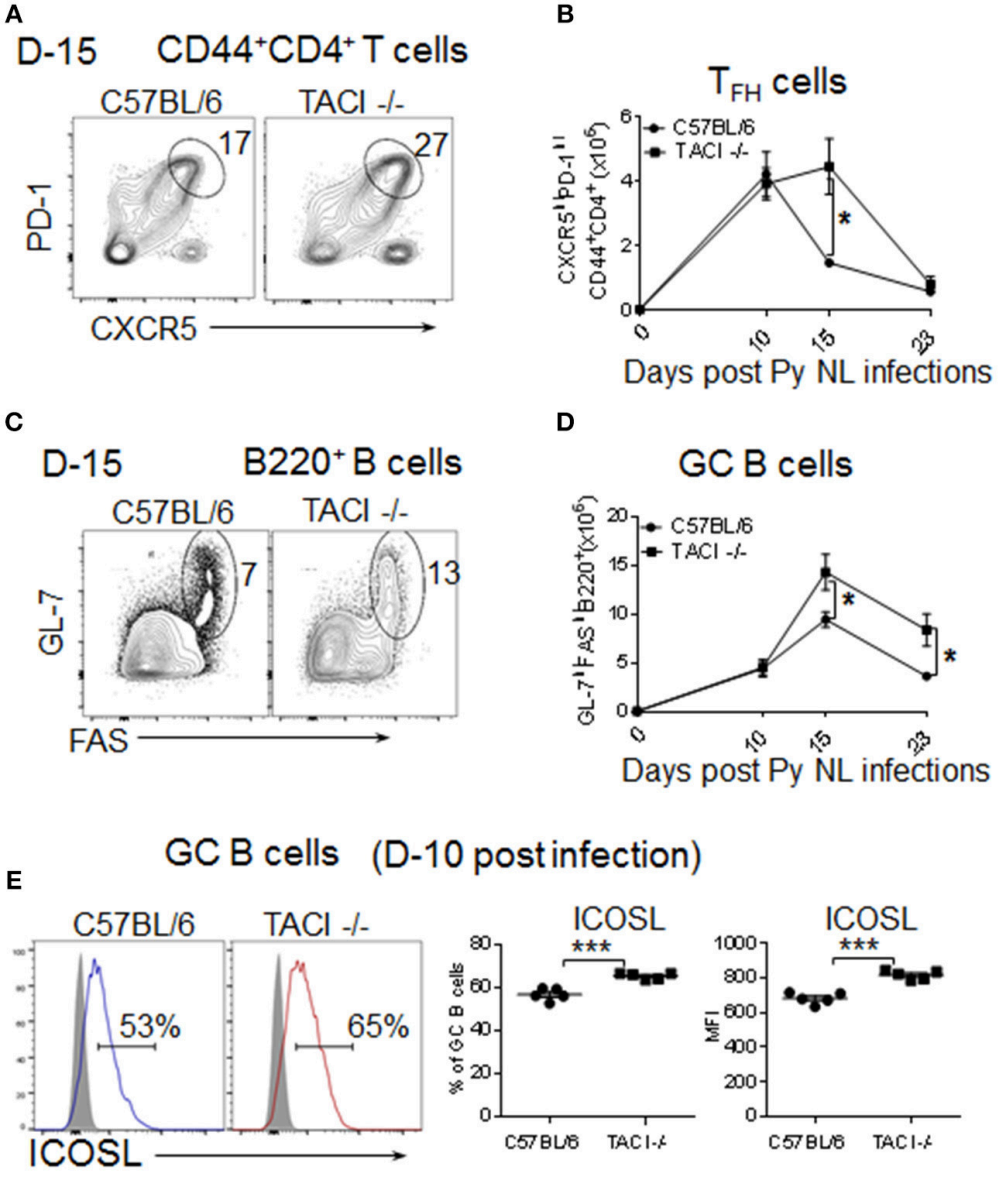
TACI deficiency extends formation and resolution kinetics of T_FH_ and GC response. TACI -/- and C57BL/6 mice were infected (i.p.) with 1 × 10^6^ *P. yoelii* (Py 17XNL strain) parasites. **(A)** Representative dot plots depict the percentage of splenic PD-1^high^CXCR5^high^ (T_FH_) cell on CD44+CD4+ pre-gated T cells at day 15 post-infection. **(B)** Formation and resolution kinetics of T_FH_ cells presented as number of T_FH_ cells per spleen. **(C)** Representative dot plots depict the percentage of and GL-7^hig^hFAS^high^ (GC B cells) on B220^+^ pre-gated B cells at day 15 post-infection. **(D)** Formation and resolution ki“netics of GC B cells presented as number of GC B cells per spleen. Total splenic B cell **(E)** Day 10 post-infection ICOSL expression levels were measured on B220^+^GL-7^high^FAS^high^ gated splenic GC B cells. Representative histograms as well as frequencies of ICOSL expressing cells and ICOSL MFI for each mouse strain are shown. Unpaired Student's *t*-test was used for statistical evaluation. Results are expressed as mean ± SEM (*n* = 5) from one representative experiment out of three with similar results. *p < 0.05 and ***p < 0.001 for TACI -/- vs. C57BL/6 comparison. GC, germinal center.

**Figure 3:**
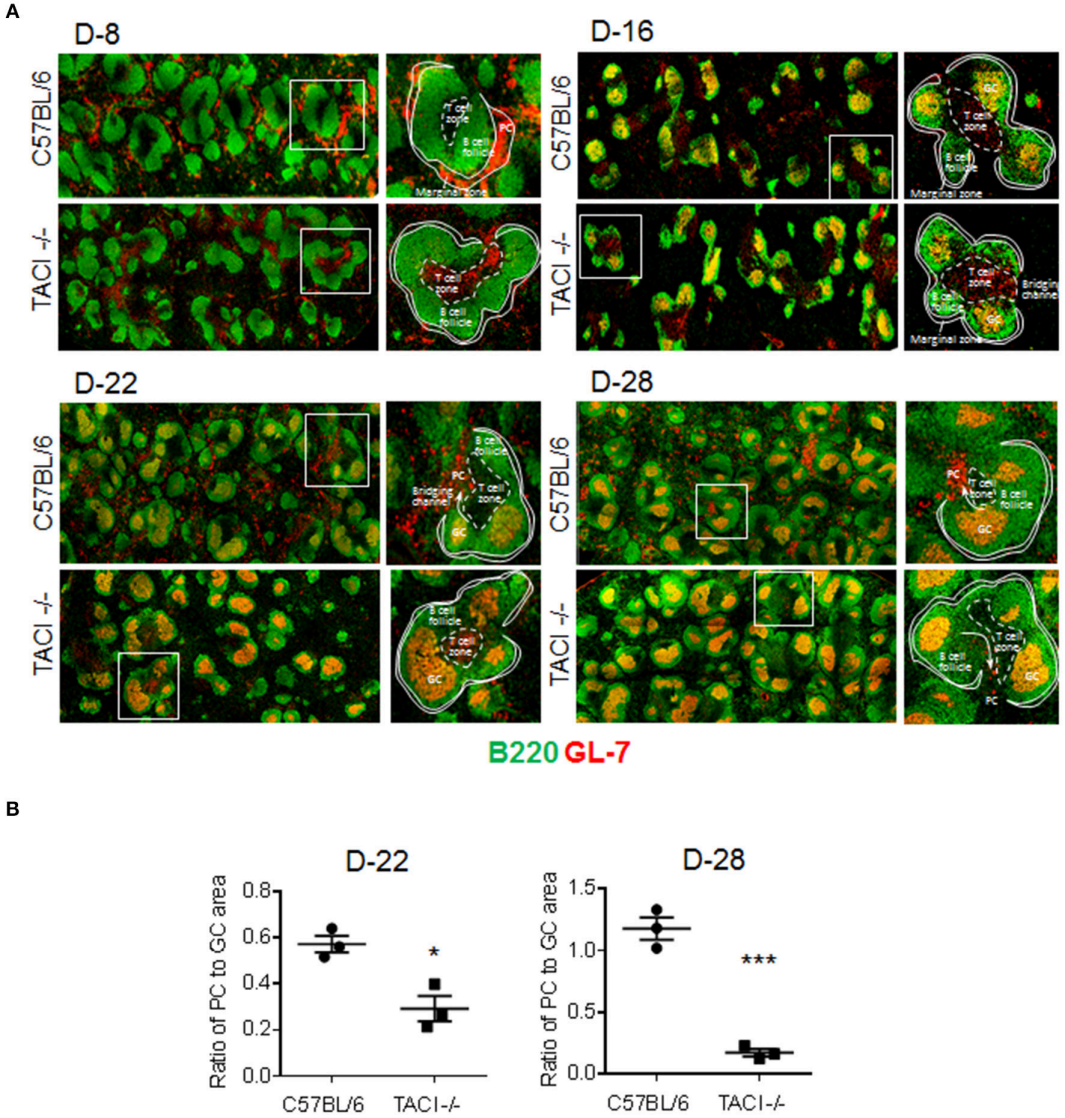
PC output from GC is limited in TACI -/- mice. TACI -/- and C57BL/6 mice were infected (i.p.) with 1 × 10^6^ *P. yoelii* (Py 17XNL) parasites. Splenic GC and PC were examined by microscopy on indicated days after infection. **(A)** Histologic sections of spleens were stained with anti-B220 (green) and anti-GL-7 (red) antibodies to visualize B-cell follicles (green), GC (B220^+^GL-7^+^, yellow), and PC (B220^low^GL-7^+^, orange). T-cell independent PC are located in the marginal zone and T-cell dependent PC in red pulp of the spleen, adjacent to the “Bridging channels.” T cell zones were defined as central location where tightly packed follicles are distributed around and the activated T cells (B220^−^GL-7^+^, red) are located. Arrows indicate the plasmablast pathway to extrafollicular areas through the “Bridging channels.” **(B)** PC output was calculated by ratio of PC foci to GCs in half spleen. Each dot represents one mouse. Magnification is 10x. *p < 0.05 and ***p < 0.001 for TACI -/- vs. C57BL/6 comparison. Experiment was performed once.

T_FH_ cells provide soluble and contact-dependent signals to B cells for somatic hypermutation and affinity maturation of antibodies in the GC (23). Following T_FH_ help, activated B cells leave the GC as plasmablasts and memory B cells (24). Moreover, the formation of T_FH_ cells and their communication with GC B cells is required for the development of parasite-specific B cells during *Plasmodium* infection (11, 25). The discovery of delayed kinetics of anti-malarial antibody development in *P. yoelii* infected TACI -/- mice led us to investigate the T_FH_ development and GC formation in infected mice spleens. In wild-type mice, the number of CD4^+^CD44^+^CXCR5^high^PD-1^high^ T_FH_ cells (Supplementary Figure 2A) were highest at day 10 post-infection, which steadily declined on days 15 and 23 (Figures 2A,B). The percentage of T_FH_ cells in wild-type mice followed kinetics similar to the number of T_FH_ cells (Supplementary Figure 2B). Compared to T_FH_ cells, wild-type mice B220^+^GL7^+^FAS^+^ (Supplementary Figure 2A) GC B cell-percentages and numbers peaked slightly later (day 15) (Figures 2C,D). In TACI -/- mice, day 10 T_FH_ cell-numbers were comparable to those of wild-type mice on the same day (Figures 3A,B). However, instead of declining thereafter as in wild-type mice, TACI -/- T_FH_ cell-numbers persisted until day 15. The kinetics of TACI -/- mice T_FH_ cell-percentage was similar to the changes in T_FH_ cell numbers (Supplementary Figure 2B). This delayed resolution of T_FH_ cells coincided with a significantly more GC B cell numbers in TACI -/- mice than wild-type mice on day 15 (Figures 2C,D). Both mouse strains had lower numbers of GC B cells on day 23 than on day 15, but TACI -/- mice cell numbers were still significantly more than those of wild-type mice. Similar to the number of GC measured on day 15, the percentage of GC B cells on the same day were significantly higher in TACI -/- mice than wild-type mice, but the difference between the two mouse strains vanished on day 23 (Supplementary Figure 2C).

Although the CD4^+^CD44^+^CXCR5^high^PD-1^high^ cells were initially designated as T_FH_ cells that promote B cell responses in the GC (23), more recent studies showed that a Foxp3-expressing regulatory subset of T_FH_ cells (T_FR_) is inhibitory to the GC reaction and B cell responses (26, 27). The function of these cells can be assessed by analyzing the ratio of T_FR_ to T_FH_ (T_FR_:T_FH_) cells, which predicts the influence of T_FR_ cells on antibody responses (28). Since we measured lower anti-parasite antibody levels despite the persistent of T_FH_ and GC in TACI -/- mice, higher T_FR_:T_FH_ ratio could be responsible for the ablated antibody responses. However, this possibility was ruled out because we measured comparable T_FR_:T_FH_ ratios between the mouse strains at 15 day time point (Supplementary Figures 3A–C).

Interaction of ICOSL on B cells with ICOS on pre-T_FH_ cells is essential for early stage T_FH_ cell formation (29). In nitrophenyl-chicken gamma globulin immunized TACI -/- mice, the elevated ICOSL expression has been implicated in expanded T_FH_ and GC formation (11, 12). Reminiscent of immunized and *C. rodentium* infected TACI -/- mice, we also detected significantly higher ICOSL expression on TACI -/- B cells as compared to wild-type mice at 10 days post-infection (Figure 2E). Thus, the elevated ICOSL on GC B cells likely contributes to the persistent T_FH_ and GC B cell interaction in *P. yoelii* infected TACI -/- mice.

### The emergence of PC is delayed in TACI -/- mice

**Figure 4:**
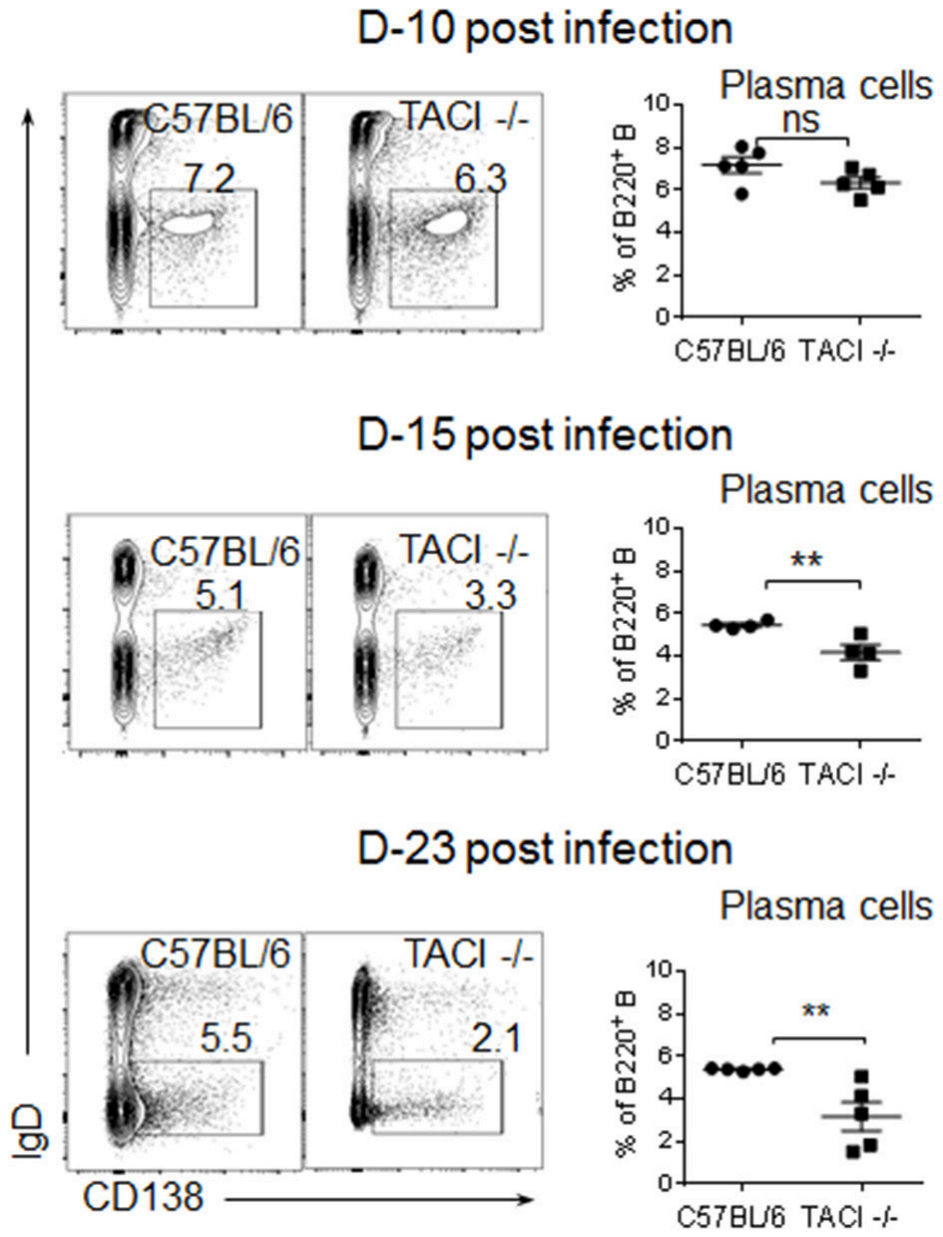
Development of PC in infected TACI -/- mice. TACI -/- and C57BL/6 mice were infected (i.p.) with 1 × 10^6^ *P. yoelii* (Py 17XNL strain) parasites. Representative dot blots of splenic B220 gated IgD-CD138^+^ plasma cells on indicated time points are shown. Mean frequencies of PC as percentages of B cells are plotted for each time point. Each data point represents a mouse. Unpaired Student's *t*-test is used for statistical evaluation. Results are expressed as mean ± SEM (*n* = 5) from one representative experiment out of two with similar results. **p < 0.01 indicates TACI -/- vs. C57BL/6 mice. ns, not significant.

Although an increase in ICOSL expression on B cells has been detected in both immunized and *C. rodentium* infected TACI -/- mice, the consequence of elevated ICOSL on PC development and antibody response was different in the two studies (11, 12). In immunized TACI -/- mice elevated ICOSL resulted in impaired PC development and reduced antibody responses (11). In contrast, *C. rodentium* infection of TACI -/- mice resulted in higher avidity antibodies that cleared the infection faster than wild-type mice did (12). To assess whether the persistence of GC for an extended duration together with increased ICOSL expression on TACI -/- GC B cells impacted the development of PC exiting the GC, we analyzed the kinetics of PC formation and GC development in the spleens of TACI -/- and wild-type mice following *P. yoelii* infection. As expected, both mice were free from GC occupying B cell follicules on day 8 post-infection (Figure 3A). We observed more PC (B220^low^GL-7^+^) located in the marginal zones of wild-type mice spleens than the marginal zones of TACI -/- mice, which suggested the initiation of T-cell independent response (30) as early as day 8 in wild-type mice because, together with B1 cells, marginal zone B cells are responsible for T-cell independent responses (31). The absence of PC (B220^low^GL-7^+^) in TACI -/- mice marginal zones is consistent with the well-established role for TACI in mediating T-cell independent responses (16, 32). At the same time, TACI -/- mice T cell zone was already populated with activated GL-7^+^ cells (33), suggesting that T cells were activated faster in TACI -/- than in wild-type mice (Figure 3A). Both strains had well-formed GC (B220^+^GL-7^+^) (34, 35) in B cell follicules as well as activated (GL-7^+^) cells occupying the T cell zone on day 16. By day 22, activated T cells were relocated to GC in the wild-type mice leaving the T cell-zones empty of GL-7^+^ T cells. Also on day 22, B220^low^GL-7^+^ PC began exiting GC from the “Bridging channels” (36) in wild-type mice (Figures 3A,B). In TACI -/- mice however, not only the population of “Bridging channels” by B220^low^GL-7^+^ PC was sparse on day 22, but also the T cell zone was still occupied with activated GL-7^+^ T cells in addition to the persistence of GCs in the follicules (Figures 3A,B). By day 28, activated T cells in the T cell zones were not present in TACI -/- spleens any longer. On the same day, the B220^low^GL-7^+^ PC in the wild-type mice “Bridging channels” were more abundant and remained significantly more than that of TACI -/- mice (Figures 3A,B). In addition to the analysis of spleens in microscopy, we also assessed the frequency of splenic IgD^−^CD138^+^ PC in *P. yoelii* infected mice in flow cytometry. Supporting the microscopy results, we found that the frequencies of PC on days 15 and 23 were significantly lower in TACI -/- mice than wild-type mice (Figure 4).

### Despite a delay in PC response, TACI -/- mice develop parasite specific ASC and high affinity antibodies following the clearance of infection

**Figure 5:**
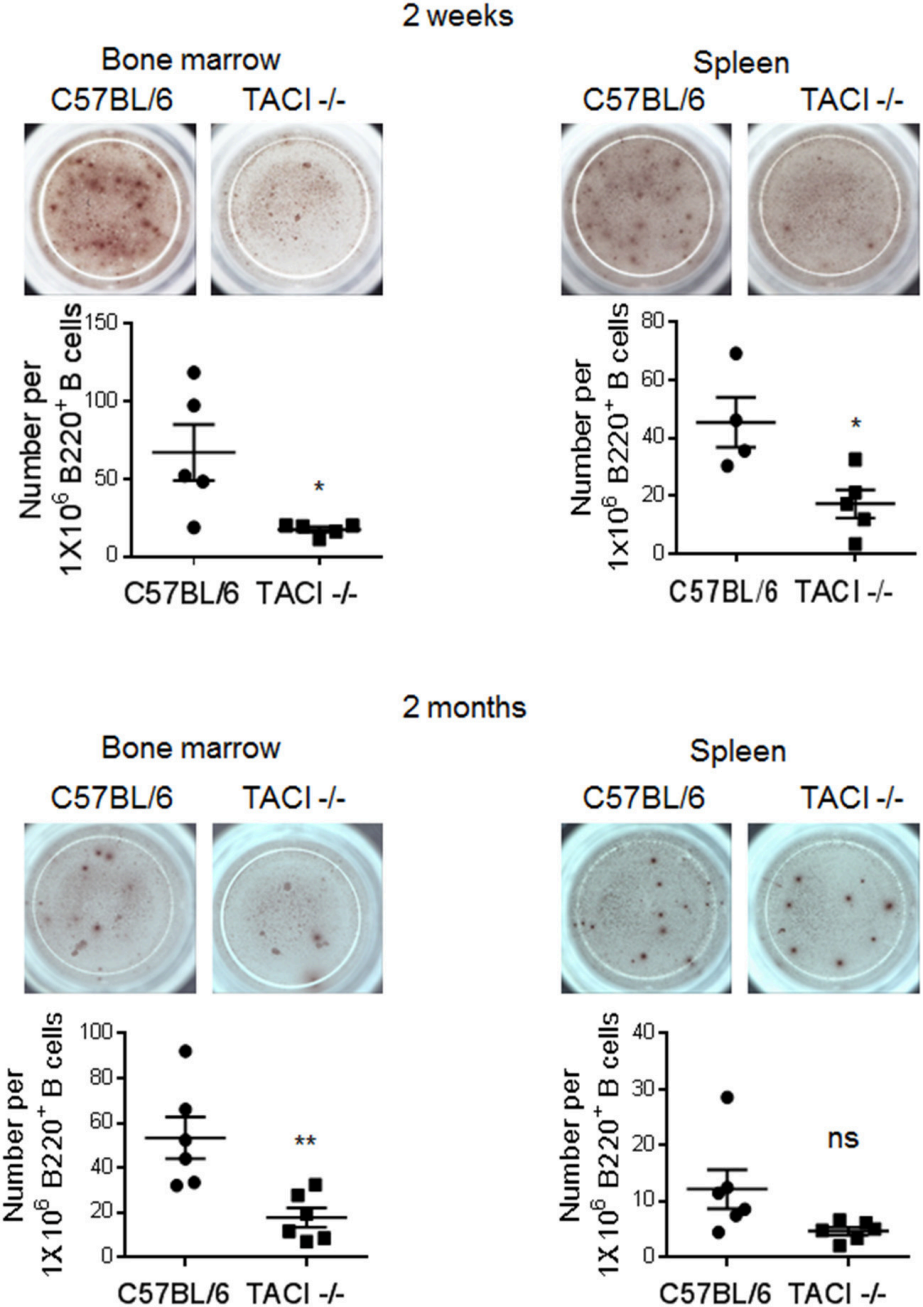
TACI deficiency causes delayed generation of rMSP-1_19_-specific ASC. Spleens and bone marrow cells were harvested 2 weeks and 2 months post-infection to examine the frequency of antigen-specific IgG antibody secreting cells (ASC) in ELISPOT assay. The number of rMSP-1_19_-specific ASC per 1 × 10^6^ B220^+^ B cells were quantified and plotted. Each data point represents one mouse. Unpaired Student's *t-*test is used for statistical evaluation. Results are expressed as mean ± SEM (*n* = 4–6) from one representative experiment out of two with similar results. *p < 0.05 and **p < 0.01 indicate TACI -/- vs. C57BL/6 mice. ns, not significant.

The discovery of significantly lower numbers of PC in TACI -/- mice as compared to wild-type mice at the time of parasite clearance (day 25), prompted us to measure *Plasmodium-*specific ASC during and after the infection. Reflecting the difference in anti-*P. yoelii* antibody levels between the two strains, we found significantly lower numbers of ASC recognizing the rMSP-1_19_ protein in the spleen and bone marrow of TACI -/- mice 2 weeks post-infection (Figure 5). At the 2-month time point, TACI -/- mouse cells were still less than the wild-type cells but only the bone marrow ASC were statistically significantly lower in TACI -/- mice. In addition to aiding in the expansion of antibody secreting GC B cells, T_FH_ cells also promote antibody affinity maturation. At 71 days post-infection, we found no difference in the avidity of antibodies directed against rMSP-1_19_ protein between the mouse strains (Supplementary Figure 4). These observations suggested that anti-*P. yoelii* antibodies secreted from splenic ASC may be responsible for the resolution of infection in TACI -/- mice.

### TACI -/- mice were protected from re-infection with *P. yoelii* parasites

**Figure 6:**
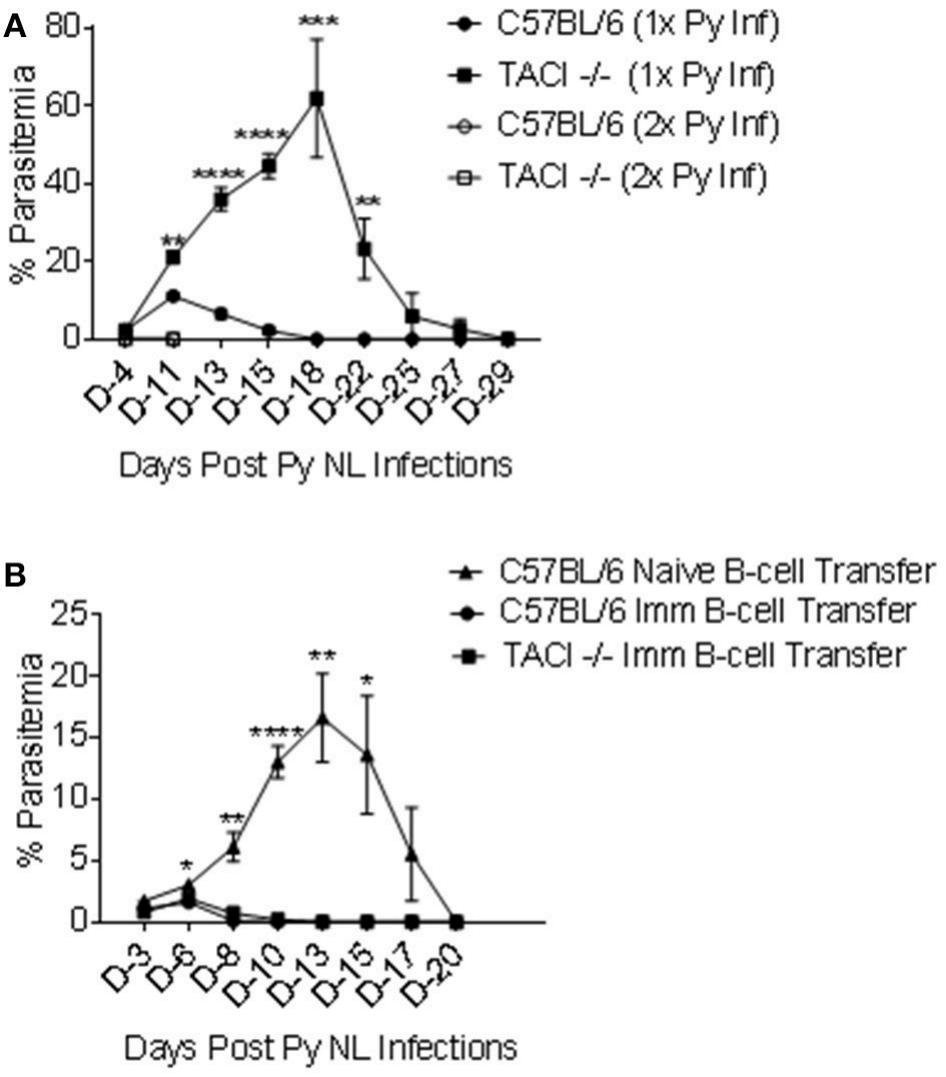
TACI -/- mice are resistant to re-infection with *P. yoelii* and *P. yoelii* experienced TACI -/- mice B cells are protective. **(A)** TACI -/- and C57BL/6 mice were re-infected (i.p.) with 1 × 10^6^ *P. yoelii* (Py 17XNL) parasites 11 months after the first infection (2x Py inf). A second group of naïve TACI -/- and C57BL/6 mice was infected for the first time to recapture the first infection parasitemia (1x Py inf). Parasitemia levels were evaluated by blood smears at indicated days post challenges. Results are expressed as mean % parasitemia ± SEM for 10 mice per group for first challenge, and for 5 mice per group for the second challenge. Unpaired student's *t-*test was used for statistical evaluation. *indicates *p* < 0.05, **p < 0.01;***p < 0.001; and ****p < 0.0001 when comparing 1x Py NL and 2x Py infected TACI -/- mice. Experiment was performed once. **(B)** B cells from naive C57BL/6 or *P. yoelii* 17XNL infected C57BL/6 and TACI -/- mice were adoptively transferred into 3 to 4 naïve C57BL/6 mice. Two hours after B-cell transfer, mice were challenged (i.p.) with 1 × 10^6^ *P. yoelii* parasites and parasitemia were assessed on indicated days. Unpaired student's *t* test was used for statistical evaluation. Results are expressed as mean % parasitemia ± SEM. *indicates *p* < 0.05, **p < 0.01;***p < 0.001; and ****p < 0.0001 when comparing the naïve B cell transfer and the TACI -/- B cell transfer groups. Experiment was performed once.

B cells are crucial for the development of protective immunity against the erythrocytic stages of *Plasmodium* (37, 38). Since TACI -/- mice eventually cleared the infection and elicited parasite specific ASC and antibodies with affinities comparable to those of wild-type mice, we assessed whether they were resistant to a second challenge as has been shown for wild-type mice (39). TACI -/- and C57BL/6 mice were infected a second time with 1 × 10^6^ *P. yoelii* 11 months after the clearance of the first infection. Naïve mice were also infected as a control. As expected, higher and delayed peak parasitemia, as well as delayed parasitemia resolution (day 29 post-infection) was observed in 1X infected TACI -/- mice as compared to wild-type mice (Figure 6A). Also, naïve wild-type mice parasitemia peaked on day 11 post-infection and was cleared by day 18. TACI -/- and C57BL/6 mice that had been infected previously were protected from a second *P. yoelii* infection, indicating a sustained long-term immunity. In these experimental groups, parasitemia levels at day 4 post-infection were 0.025% and 0.023% for TACI -/- and C57BL/6 mice, respectively, while no parasites were detected at day 11-time point.

## Adoptive transfer of *P. yoelii* immune B-cells afford protection

The association between antibody development and parasite clearance in both the strains suggested that the resistance to a second infection is likely a result of the persistence of B cell memory and/or PC. In a cerebral malaria model, CD19^+^ B cells from *Plasmodium berghei* recovered mice confers protection in naïve mice (40). To assess the role of parasite experienced B cells in the protection of TACI -/- mice against *P. yoelii* infection, we performed adoptive transfer experiments. Naïve B cells from C57BL/6 mice or B cells from previously infected C57BL/6 and TACI -/- mice (4 months earlier) were used as donor cells. Thirty million B cells were adoptively transferred into each naïve C57BL/6 mouse two hours before the experimental challenge with 1 × 10^6^ *P. yoelii* parasites. In mice receiving naïve B cells, peak parasitemia level and time-point (day 13) (Figure 6B) were comparable to those of naïve mice receiving *P. yoelii* for the first time (Figure 1A). In contrast, C57BL/6 mice that were adoptively transferred with *P. yoelii* experienced cells from either strain had very low peak parasitemia levels (day 6), which resolved by day 10 post-infection in both mouse strains (Figure 6B). Thus, the B cell immunity developed after *P. yoelii* infection of TACI -/- mice is transferable and sufficient to render naïve mice resistant to *P. yoelii* challenge.

## Discussion

Infant susceptibility to malaria is likely compounded by the unique features of their immune system. Both the qualitative and quantitative antibody responses to malaria are tightly associated with the age of infected infants (41–43). Incomplete understanding of the underlying molecular and cellular requirements supporting the development and maintenance of long lasting anti-malarial memory B cell and PC responses in infants hamper the development of effective malaria vaccines in children. In this study, we investigated the role of TACI in resistance to malaria infection because TACI is important for the development and maintenance of PC (5, 8–10, 15).

Both human and mouse studies suggested a role for the TACI ligand BAFF in host response to malaria infection. Elevated serum BAFF has been measured in adults experimentally infected with *P. falciparum* and in children with acute malaria (13, 14). The increase in serum BAFF levels is accompanied by a decrease in BAFF-R and an increase in TACI and BCMA on B cells of malaria-infected children (13). Despite these reports, the contribution of TACI in malaria infection has not been explored. We have previously shown that low TACI expression in neonatal B cells is responsible for the inability of this age group to respond to polysaccharide vaccines (15). We hypothesized that low TACI expression might also promote the suboptimal anti-malarial humoral immune response observed in infants. Significantly elevated levels and the delayed resolution of parasitemia in *P. yoelii* infected TACI -/- mice indicated that TACI is important for controlling malaria. Elicited protective humoral immunity was likely responsible for the clearance of *Plasmodium* in TACI -/- mice because the delayed clearance of *P. yoelii* coincided with the late emergence of anti-malaria antibodies, and B cells transferred from *P. yoelii* immune TACI -/- mice afforded protection in naïve wild-type mice, and TACI -/- mice rechallenged with *P. yoelii* resisted infection. Moreover, recovered TACI -/- mouse spleens contained *P. yoelii* specific ASC with comparable antibody avidity to those secreted from wild-type mice. Both T-cell dependent and independent mechanisms are involved in the development of anti-malaria antibodies (30). Since TACI expression is required for the development of T cell independent antibody responses, impairment in the antibodies targeting malarial T cell independent antigens likely have contributed to delayed parasitemia resolution. Indeed, we detected the appearance of GL7^+^ PC in the splenic marginal zones of wild-type mice as early as day 8 after infection while the TACI -/- mice marginal zone PC response was absent. Additional association between TACI expression and TI B cell responses to malaria may be related to the effect of toll-like receptor (TLR) 9 on TACI expression. We have previously shown that the TLR9 agonist CpG boosts BAFF and APRIL mediated PC generation by strongly upregulating the expression of TACI on B cells (44). Since, TLR9 deficiency compromises host control of parasitemia (45), TACI deficiency may also be negating the beneficial effect mediated by malaria TLR9 agonists (46).

Previous reports have shown that the formation of T_FH_ and GC is amplified in TACI -/- mice after immunization (11) and infection (12). However, while the increased T_FH_ and GC development was beneficial in eliciting high affinity antibodies that helped resist *C. rodentium* infection (12), nitrophenyl-chicken gamma globulin immunized TACI -/- mice generated lower levels of nitrophenyl-specific antibodies with diminished affinities (11). The immunization study also demonstrated an ablated ASC development in TACI -/- mice, which was attributed to the decrease in PC survival, a likely consequence of the absence of TACI mediated survival signals (11). Diminished ASC maintenance was reported in another study where influenza infected TACI -/- mice developed lower levels of virus-specific ASC compared to wild-type mice (10). Like the previous reports (11, 12), we observed exaggerated magnitude and kinetics in TACI -/- mice T_FH_ and GC formation after *Plasmodium* infection. Despite the augmented T_FH_ and GC B responses, TACI -/- mice were not able to control the parasitemia partly due to the delay in the emergence of PC from GC and the development of parasite specific ASC. Although the increased expression of ICOSL may be contributing to the persistence of Tfh and GC B cells, it can also be a result of persistent stimulation with parasite antigens as has been shown in a model where boosting with peptide stimulates T_FH_ response without increasing B cell response (47).

Supporting the clinical observations where high level of BAFF is measured in *Plasmodium* infected individuals (13, 14), stimulation of monocytes with soluble *Plasmodium* molecules, soluble schizont fraction of Plasmodium falciparum antigen (sPFAg) and hemozoin (HZ) both induce the expression of BAFF (48). Although these studies suggested a possible involvement of increased serum BAFF in the activation of B cells and generation of antibody secreting cells in malaria, by analyzing *P. yoelii* infected mice, Liu et al detected a decrease in the number of dendritic cells (DCs) expressing membrane BAFF after malaria challenge (49). Since multimeric BAFF, but not trimeric serum BAFF, is able to promote PC by engaging TACI (50), the authors proposed a possible link between the disappearance of malaria specific ASC with the decrease in BAFF expressing DCs in malaria endemic regions. It remains to be seen whether the decrease in BAFF expressing DCs is accompanied by a decrease in the other TACI ligand, APRIL, and if not, whether APRIL can or cannot compensate for the diminished BAFF expressing DCs in sustaining the survival of ASC. Regardless of the significance of the changes in BAFF and APRIL expression, our study highlights the importance of TACI mediated development of ASC in controlling malaria infection. Interestingly, despite the delay in the generation of malaria specific antibodies and recovery from infection, TACI deficient mice remained resistant to second malaria challenge even after 11 months. Malaria specific B cells elicited in TACI -/- mice were not only able to clear the infection but also could render naïve mice resistant to malaria challenge.

Our study highlights the importance of TACI mediated control of T_FH_ and GC response, and ASC development during malaria infection. These findings may have implications in understanding the immunobiological bases of infant-susceptibility to malaria since TACI expression is severely reduced in neonatal B cells in mice and in humans (15, 51). Moreover, as in TACI -/- mice, the development of humoral immune response is delayed in children from malaria endemic area (4, 52). Further work is needed in infant populations and in murine models of neonatal malaria to elucidate the impact of low TACI expression on the phenotype of T_FH_ cells during malaria infection and vaccine response.

## Author contributions

MA, MP, and JY: designed the study and wrote the manuscript; MP, JY, MW, SD, AY, TS, and BS: performed the experiments; MP, JY, MW, SD, AY, TS, BS, AM, and MA: analyzed data.

### Conflict of interest statement

The authors declare that the research was conducted in the absence of any commercial or financial relationships that could be construed as a potential conflict of interest.
