# Supplementary material for: TACI Contributes to Plasmodium yoelii Host Resistance by Controlling T Follicular Helper Cell Response and Germinal Center Formation
Source: Front Immunol. 2018 Nov 9;9:2612. doi: 10.3389/fimmu.2018.02612 (PMC6237915; doi:10.3389/fimmu.2018.02612)
Supplement: Supplementary file 1 [file Data_Sheet_1.docx]

Supplemental figures

TACI contributes to *Plasmodium yoelii* host resistance by controlling T follicular helper cell response and germinal center formation

Marcela Parra, Jiyeon Yang, Megan Weitner, Steven Derrick, Amy Yang, Thomas Schmidt, Balwan Singh, Alberto Moreno, and Mustafa Akkoyunlu

**Supplemental Figure 1**

**A)**

**B)**

**Anti-*P. yoelii* antibody development is delayed in TACI -/- mice.** TACI -/- and C57BL/6 mice were infected (i.p) with 1 x 10^6^ *P. yoelii* 17XNL parasites. Pooled sera from each strain (3 mice per time point) on days 8,16, 22 and 28 post parasite challenge were tested in ELISA for anti-rMSP-1_19_ (A) anti-*P yoelii* IgG (B) antibody measurement. ELISA negative cut-off value was determined by 3-fold OD values from wells containing sera from naïve mice. The endpoint titer of the given sample was defined as the reciprocal of the last dilution with OD value greater than of the cut-off value 0.036 for A and 0.091 for B. *Represents endpoint titer for TACI -/- and & for C57BL/6 mice. Experiment was performed once.

**Supplemental Figure 2**


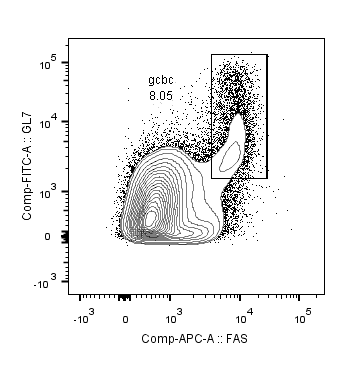

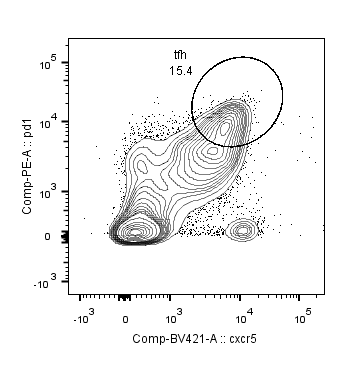


FAS

GL-7

CXCR5


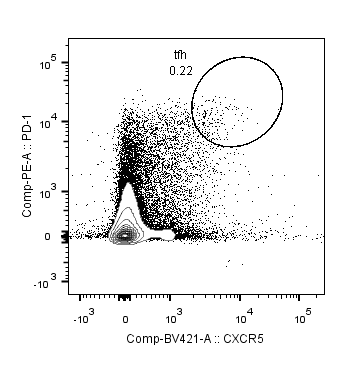

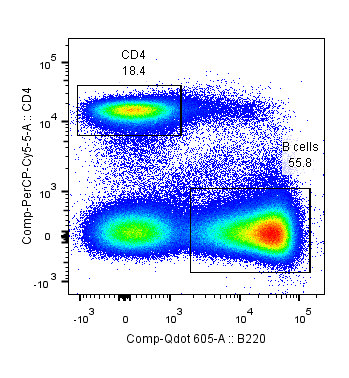

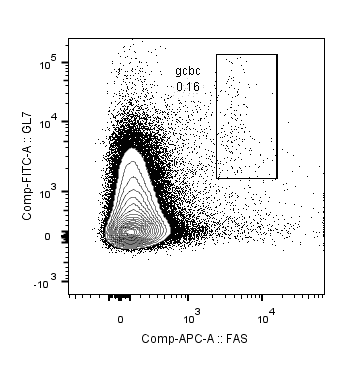


Lymphocyte

CD44^+^CD4^+^ TC

B220^+^ BC

B220

PD-1

0.22

15.4

T_FH_

T_FH_

0.16

GC BC

GC BC

8.05

Naive

Parasite

infection

Naive

Parasite

infection


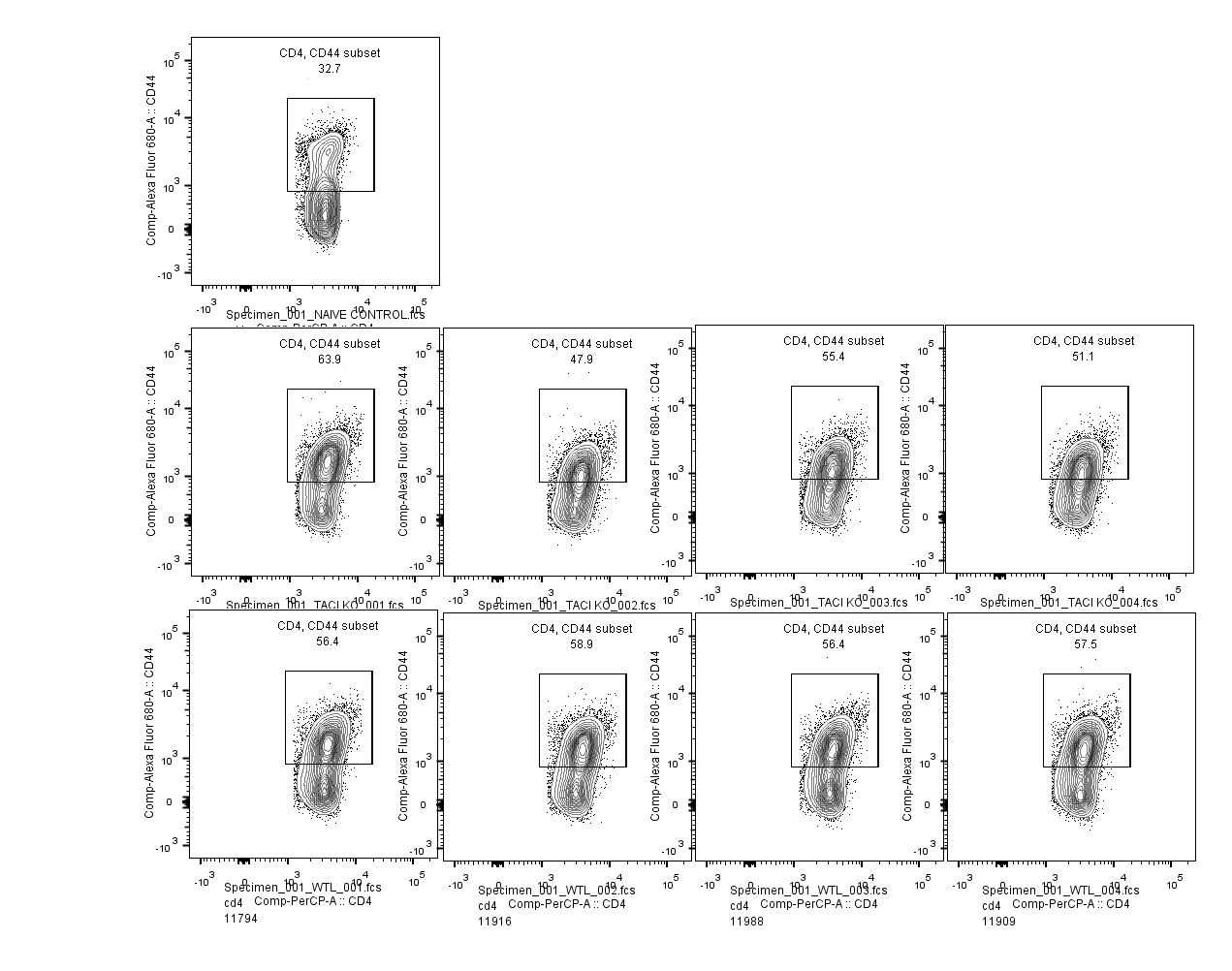


CD4

CD44

CD4


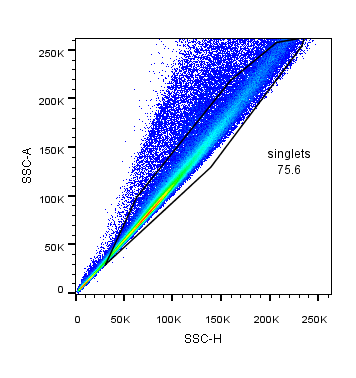

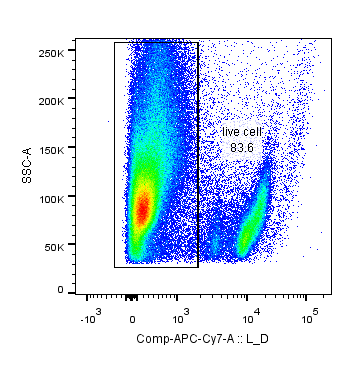

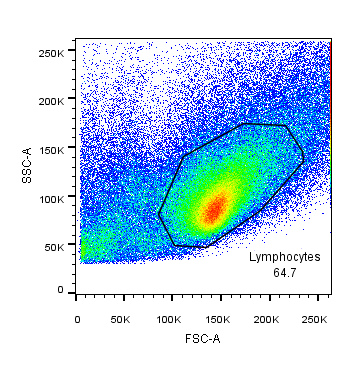


SSC-A

SSC-H

Live/dead

FSC

Singlets

Live cells

**A)**

**C)**

**B)**

Days post Py NL infections

T_FH_ Cells

*

*

Days post Py NL infections

GC B cells

**Percentages of splenic T_FH_ and GC populations in *P. yoelii* infected mice.** Spleens were harvested at indicated days post infection for flow cytometry analysis. Gating strategies for CD44^+^CD4^+^ pre-gated PD-1^+^CXCR5^+^ (T_FH_) cells and B220^+^ pre-gated GL-7^+^FAS^+^ (GC) B cells in naïve and parasite infected mice are shown (A). Formation and resolution kinetics of (T_FH_) cells (B) and GC B cells (C) are presented as percentages of cells per spleen. Results are expressed as mean ± SEM (n=5) from one representative experiment out of three with similar results. *p<0.05 depicts statistical significance between TACI -/- and C57BL/6 mice.

**Supplemental Figure 3**

**B)**

T_FR_ cells

**A)**


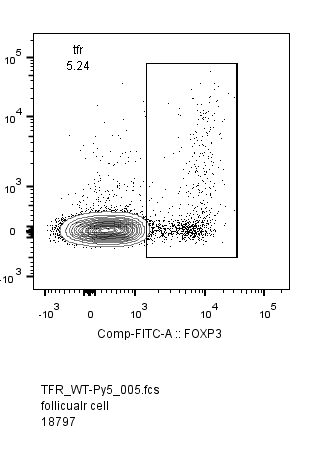

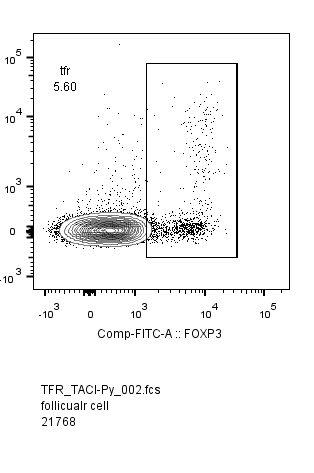


5.2

5.6

C57BL/6

TAC I ^-/-^

T_FH_ cells

***

Tfr

Tfr

CD25

Foxp3

ns

ns

**C)**

**Percentages of splenic** **T_FR_ cell populations in *P. yoelii* infected mice.** Spleens were harvested on day 15 post infection for flow cytometry analysis. (A) Average PD-1^+^CXCR5^+^ (T_FH_) cell percentages are plotted. (B) Representative dot plots depict Foxp3+ T_FR_ cells among CD4^+^CD44^+^PD-1^+^CXCR5^+^ cells. (C) Average percentage of T_FR_ cells among T_FH_ cell population as well as the number of T_FR_ cells per spleen are plotted. Results are expressed as mean ± SEM (n=5) from one representative experiment out of three with similar results. ***p<0.05 depicts statistical significance between TACI -/- and C57BL/6 mice. ns: not significant. Unpaired Student’s t-test was used for statistical evaluation.

**Supplemental Figure 4**

ns

**Average avidity Index ratio of IgG antibodies against recombinant MSP-1_19_ for C57BL/6 and TACI -/- mice.** Sera were collected from 5 C57BL/6 and 5 TACI -/- mice 71 days post *P. yoelii* infection. Samples were treated with 0.1M GuHCl as a dissociative agent or with PBS/0.05% Tween for the controls. The avidity index ratio was calculated as (AUC of the guanidine hydrochloride-treated samples) / (AUC for the PBS-treated controls) for each serum titration. The avidity index mean is represented as the mean of three avidity index ratios ± SEM. ns: not significant. Experiment was performed once.
